# Prevalence and management of ectopic and molar pregnancies in 17 countries in Africa and Latin America and the Caribbean: a secondary analysis of the WHO multi-country cross-sectional survey on abortion

**DOI:** 10.1136/bmjopen-2024-086723

**Published:** 2024-10-14

**Authors:** Camila Ayume Amano Cavalari, Hedieh Mehrtash, Vanessa Brizuela, Adama Baguiya, Kwame Adu-Bonsaffoh, Jose Guilherme Cecatti, Luis Bahamondes, Charles M'poca Charles, Philip Govule, Jean-Paul Dossou, Renato T Souza, Luis Henrique Leão, Veronique Filippi, Özge Tunçalp, Luiz Francisco Baccaro

**Affiliations:** 1 Departamento de Tocoginecologia, Universidade Estadual de Campinas Faculdade de Ciencias Medicas, Campinas, Sao Paulo, Brazil; 2 UNDP/UNFPA/UNICEF/WHO/World Bank Special Programme of Research, Development and Research Training in Human Reproduction (HRP), Department of Sexual and Reproductive Health and Research, World Health Organization, Geneve, Switzerland; 3 Kaya Health and Demographic Surveillance System (Kaya-HDSS), Ouagadougou, Burkina Faso; 4 Department of Obstetrics and Gynecology, Korle Bu Teaching Hospital, Accra, Greater Accra, Ghana; 5 Epidemiology and Disease Control, University of Ghana College of Health Sciences, Accra, Greater Accra, Ghana; 6 Health Sciences, Uganda Martyrs University Faculty of Health Sciences, Kampala, Uganda; 7 CNHU-HKM Centre de Recherche en Reproduction Humaine et en Démographie, Cotonou, Benin; 8 Public Health, Instituut voor Tropische Geneeskunde, Antwerpen, Flanders, Belgium; 9 London School of Hygiene & Tropical Medicine, London, UK

**Keywords:** OBSTETRICS, GYNAECOLOGY, Hospitals, Public

## Abstract

**Abstract:**

**Introduction:**

There are limited global data on ectopic pregnancy (EP) and molar pregnancy (MP), making it important to understand their epidemiology and management across different regions. Our study aimed to describe their prevalence for both conditions, severity of their complications and management among women in selected health facilities across 17 countries in Africa and Latin America and the Caribbean (LAC).

**Methods:**

This is a secondary analysis of the WHO multi-country survey on abortion. Data were collected from 280 healthcare facilities across 11 countries in Africa and 6 in LAC. Sociodemographic information, signs and symptoms, management and clinical outcomes were extracted from medical records. Facility-level data on post-abortion care (PAC) capabilities were also collected, and facilities were classified accordingly. χ^2^ or Fisher’s exact tests were used to compare categorical data.

**Results:**

The total number of women with EP and MP across both regions was 9.9% (2 415/24 424) where EP accounted for 7.8% (1 904/24 424) and MP for 2.1% (511/24 424). EP presented a higher severity of complications than MP. At admission, 49.8% of EP had signs of peritoneal irritation. The most common surgical management for EP was laparotomy (87.2%) and for MP, uterine evacuation (89.8%). Facilities with higher scores in infrastructure and capability to provide PAC more frequently provided minimal invasive management using methotrexate/other medical treatment (34.9%) and laparoscopy (5.1%).

**Conclusion:**

In Africa and LAC, EP and MP cause significant maternal morbidity and mortality. The disparity in the provision of good quality care highlights the need to strengthen the implementation of evidence-based recommendations in the clinical and surgical management of EP and MP.

**Strengths and Limitations of this study**

- We used a standardised approach to identify the prevalence and management of ectopic pregnancy (EP) and molar pregnancy (MP) across a diverse range of facilities in 17 countries.
- The large sample size and number of participating facilities enable generalisability of results to facilities with similar outcomes and geographical areas to those included in our study and other sub-Saharan African and Latin American and Caribbean countries.
- Despite implementing standardised definitions for complications, issues with medical record quality (eg, poor record keeping) may have led to underestimation and/or misclassification.
- Variations in treatment protocols across countries may have led to inconsistencies in management outcomes.
- This was a cross-sectional study; hence, we were unable to assess the long-term consequences of follow-up care of EP and MP, and therefore, their overall outcome.

## Introduction

 Ectopic pregnancy (EP) and molar pregnancy (MP) are potentially severe complications of early pregnancies, which can lead to severe maternal morbidity and mortality, especially if not diagnosed and treated early.^1^ According to the latest estimates between 1990 and 2019, EP is estimated to affect around 9.69 per 100 000 persons globally. Despite this overall improvement, certain regions like sub-Saharan Africa still experience more than other regions.^2 3^ Currently, there is an increase in EP due to a higher prevalence of pelvic inflammatory disease, assisted reproductive procedures and smoking among reproductive-age women.^4^,^6^ It is also overlooked as a cause of severe maternal morbidity and, therefore, often not included in evidence-based intervention protocols.^7^ Some studies suggested that severe outcomes are associated not only with women’s clinical characteristics but also with the quality of care provided.^8 9^

Before the 1980s, most cases of EP were diagnosed after the rupture of the trophoblastic implantation site, which was associated with acute abdominal haemorrhage, shock and increased risk of maternal death. A US retrospective study found that between 1980 and 2007, there was a decrease of approximately 50% in mortality due to a change in the natural history of EP probably due to the expanded access to early diagnosis and treatment.^10^ It is believed that in recent years, a higher proportion of EP is diagnosed at early stages before rupture; however, most data on EP come from high-income countries, and the scenario may not be the same in low and middle income countries (LMIC).^11 12^

Another important gestational complication is MP. It originates from the placenta and can potentially become a malignant neoplasm with local spread and distant metastases. Because of its low frequency and regional variations, its true incidence is difficult to estimate.^13 14^ High-income countries report MP ratios from 66 to 121 cases per 100 000 pregnancies, whereas LMICs report ratios from 23 to 1299 cases per 100 000 pregnancies.^15^ Advanced or early maternal age and a history of previous MP were identified as risk factors.^15 16^ The most common clinical picture is vaginal bleeding and a discrepancy between uterine volume and gestational age. A high level of serum beta-human chorionic gonadotropin (hCG) is another indicator of MP and can lead to consequences such as large theca lutein cysts, hyperthyroidism and early pre-eclampsia. In recent years, the clinical history of MP has also changed because of the increased availability of early ultrasonographic examination and quantitative measurement of serum hCG, leading to early diagnosis.^17 18^

Knowledge of the epidemiology of the complications of EP and MP and their management adopted in different contexts may be especially important in improving the quality of care for these conditions. However, there is a paucity of data and limited literature on EP and MP in low- and middle-income countries. The aim of our analysis was to describe the prevalence of EP and MP among women treated for early pregnancy losses, the severity of the complications and the choice of management, including facility capabilities to provide care, among women from 17 countries across Africa and Latin America and the Caribbean (LAC).

## Materials and methods

### Patient and public involvement

Patients or the public were not involved in the design, or conduct, or reporting, or dissemination plans of our research.

### Study design and subjects

Our study is a secondary analysis of the WHO multi-country survey on abortion (WHOMCS-A) focusing on women diagnosed with EP or MP. The WHOMCS-A was a large cross-sectional study with data collected in 280 healthcare facilities from 17 countries in Africa and LAC on early pregnancy loss. Data collection occurred between February 2017 and January 2019. The study protocol and main findings have already been published, detailing recruitment and collection methodologies.^19^,^21^ In each health facility, data were gathered over a 3-month period. Medical records of all women who sought care for complications related to early pregnancy loss (such as abortion, miscarriage, EP and MP) were retrospectively reviewed, and the information was entered into an online system. For the present study, we only included women with EP and MP. Data included sociodemographic information such as age, marital status, education level, obstetric history, signs and symptoms of complications related to early pregnancy loss, medical procedures performed and clinical outcome. Furthermore, facility-level data were also provided by each facility administration or a healthcare professional responsible for gynaecology and obstetrics care.

### Study measures

Women were categorised into three groups based on the severity of the complications.^19^,^22^ These categories were severe maternal outcome (SMO: maternal deaths and near miss based on the WHO definition), potentially life-threatening complication (PLTC: severe haemorrhage, systemic infection or uterine perforation) and mild/moderate complications (abnormal physical examination findings in vital signs, appearance, mental status, abdominal examination, gynaecological examination; bleeding, suspected intra-abdominal injury or infection).^22^

According to the health facility’s infrastructure and capability to provide post-abortion care (PAC; considering available treatments, personnel, diagnostic methods and specialised care), a score of facility capability to provide PAC was created.^23^ Briefly, categories were defined as facility infrastructure (FIS), standard comprehensive capability for PAC (SCPAC) and extended comprehensive capability for PAC (ECPAC). A description of post-abortion signal functions used to develop the score is in online supplemental table 1. Each score was classified as either low, intermediate or high.^23^

### Data analysis

First, we describe the prevalence of EP and MP by region (Africa or LAC) in the study sample and women’s characteristics such as marital status (dichotomized to living with or without partner); education level (no education, primary/secondary (any education until complete secondary education) and tertiary education (from incomplete tertiary education to higher education levels)); age; and number of previous births. Second, EP and MP were compared by region and PAC score according to their severity of complications. Lastly, the type of management as recorded across all women who had EP and MP was described and compared. Types of management were subdivided into surgical and clinical treatment. Surgical treatments listed were uterine evacuation (dilation and curettage, manual aspiration or both), laparotomy, laparoscopy and hysterectomy. Clinical treatments were use of a form of medical treatment (methotrexate or another form), uterotonics (misoprostol, oxytocin, ergometrine, methylergonovine and others), intravenous fluids, vasopressors, antibiotics, procoagulant agents, blood transfusion, intensive care unit (ICU) admission and prolonged facility stay (> 3 days). The analysis was then stratified by severity of complication, region and according to PAC score categories.

For the comparison of categorical variables, χ^2^ or Fisher’s exact tests were used (for expected values < 5). Statistical Analysis System (SAS) for Windows, V.9.2., SAS Institute Inc, 2002–2008, Cary, NC was used for statistical analysis.

## Results

The total number of women included in the WHOMCS-A database was 24 424, with 15 565 from Africa and 8859 from LAC. The total number of women with EP and MP across the database was 9.9% (2 415/24 424) where EP accounted for 7.8% (1 904/24 424) and MP for 2.1% (511/24 424). EP and MP were more frequent in Africa (EP 1 411/15 565, 9.1% and MP 371/15 565, 2.4%) than in LAC (EP 493/8859, 5.6%; and MP 140/8859, 1.6%) (online supplemental figure 1).

**Figure 1:**
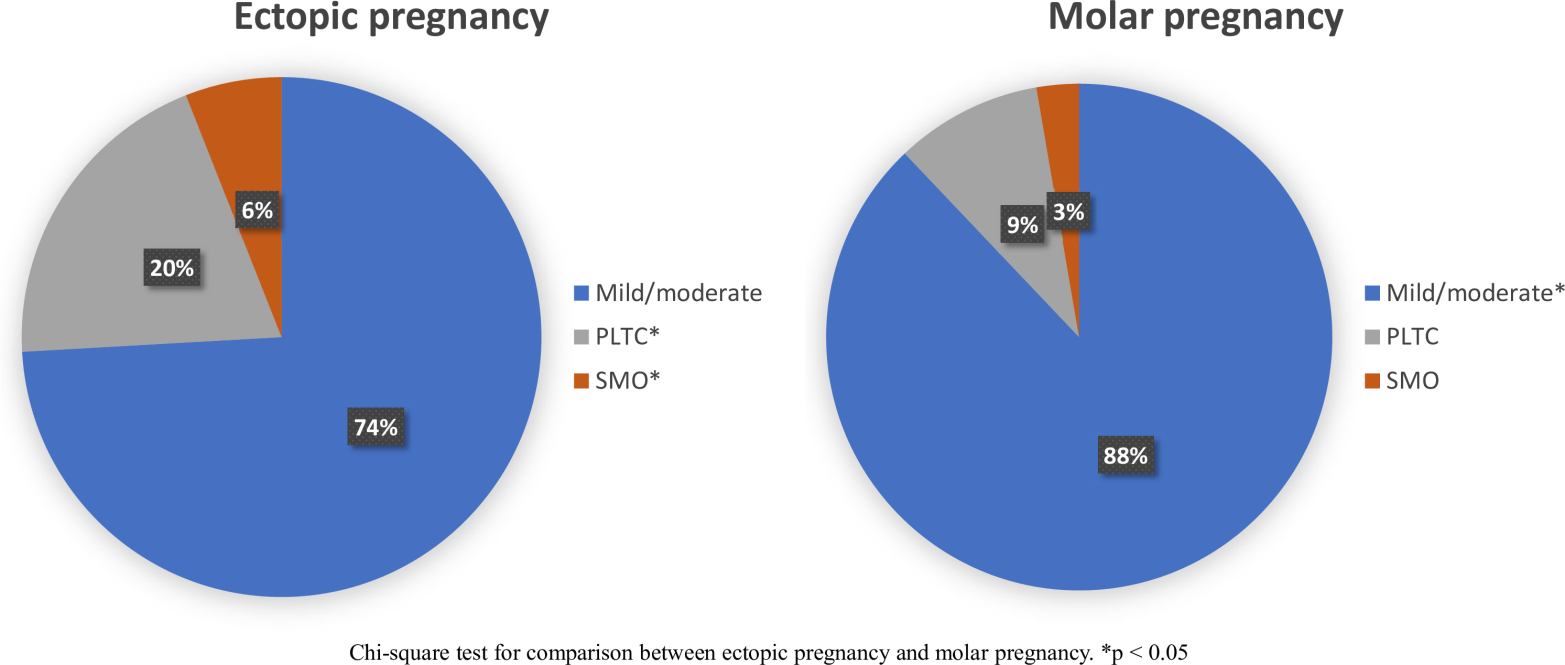
Severity of complications in ectopic pregnancy (n=1904) and molar pregnancy (n=511). PLTC, potentially life-threatening complications; SMO, severe maternal outcome.

When comparing the severity of complications between EP and MP, we observed a higher prevalence of PLTC (379/1904, 19.9%) and SMO (115/1904, 6%) in cases of EP than in cases of MP (PLTC 48/511, 9.4% and SMO 14/511, 2.7%) (figure 1).

Although most of the women with EP and MP had mild/moderate complications, among those with EP, 115/1904 (6.0%) SMO were observed (105/1904, 5.51% near misses and 10/1904, 0.52% deaths) and among those with MP, 14/511 (2.7%) SMO were observed (11/511 (2.2%) near miss and 3/511 (0.58%) deaths). Most of the women with EP presented with a lower gestational age (89.5% with < 12 weeks gestation) (table 1). Further details regarding sociodemographic characteristics by severity of complications for EP and MP are shown in online supplemental table 2.

**Table 1:** Characteristics of the analytic sample, by ectopic and molar pregnancies including region, facility infrastructure, severity, health facilities’ capability to provide PAC, clinical and sociodemographic characteristics (n=2415)

	Ectopic pregnancies (n=1904)	Molar pregnancies (n=511)
	n (%)	n (%)
Region*	1904	511
Africa	1411 (74.1)	371 (72.6)
Latin America	493 (25.9)	140 (27.4)
Facility infrastructure score	1888	508
Low	15 (0.9)	10 (2.0)
Intermediate	267 (14.1)	92 (18.1)
High	1606 (85.0)	406 (79.9)
Standard comprehensive capability for PAC score	1904	511
Low	72 (3.8)	17 (3.3)
Intermediate	867 (45.5)	111 (21.7)
High	965 (50.7)	383 (75.0)
Extended comprehensive capability for PAC score	1904	511
Low	35 (1.8)	11 (2.2)
Intermediate	1337 (70.2)	322 (63.0)
High	532 (28.0)	178 (34.8)
Severity	1904	511
Severe maternal outcomes	115 (6.0)	14 (2.7)
Potentially life-threatening complications	379 (19.9)	48 (9.4)
Mild/moderate	1410 (74.1)	449 (87.9)
Age	1885	502
<20	100 (5.3)	70 (13.9)
20–24	425 (22.5)	130 (25.9)
25–29	568 (30.1)	97 (19.3)
30–34	466 (24.7)	70 (13.9)
≥35	326 (17.3)	135 (26.9)
Cohabitation status	1767	489
With partner	1296 (73.3)	399 (81.6)
Without partner	471 (26.7)	90 (18.4)
Number of previous births	1473	387
0	162 (11.0)	21 (5.4)
1–2	942 (64.0)	179 (46.3)
>2	369 (25.0)	187 (48.3)
Gestational age	1396	348
<12	1249 (89.5)	116 (33.3)
≥12	147 (10.5)	232 (66.7)
Education	1476	410
No education	163 (11.0)	106 (25.9)
Primary/secondary	981 (66.5)	264 (64.4)
Tertiary	332 (22.5)	40 (9.7)

* Participating countries for Africa: Benin, Burkina Faso, Chad, Democratic Republic of the Congo, Ghana, Kenya, Malawi, Mozambique, Niger, Nigeria, Uganda; participating countries for LAC: Argentina, Bolivia, Brazil, Dominican Republic, El Salvador and Peru.

LACLatin America and the CaribbeanPACpost-abortion care

At facility admission, most women with EP had abdominal pain (91.7%), 66% had vaginal bleeding and 49.8% had signs of peritoneal irritation. Among women with MP, 82.3% had vaginal bleeding, 53.9% had a uterine volume greater than expected for the gestational age and 24.8% had vomiting. Details of signs and symptoms at facility admission are shown in the online supplemental table 3.

**Figure 2:**
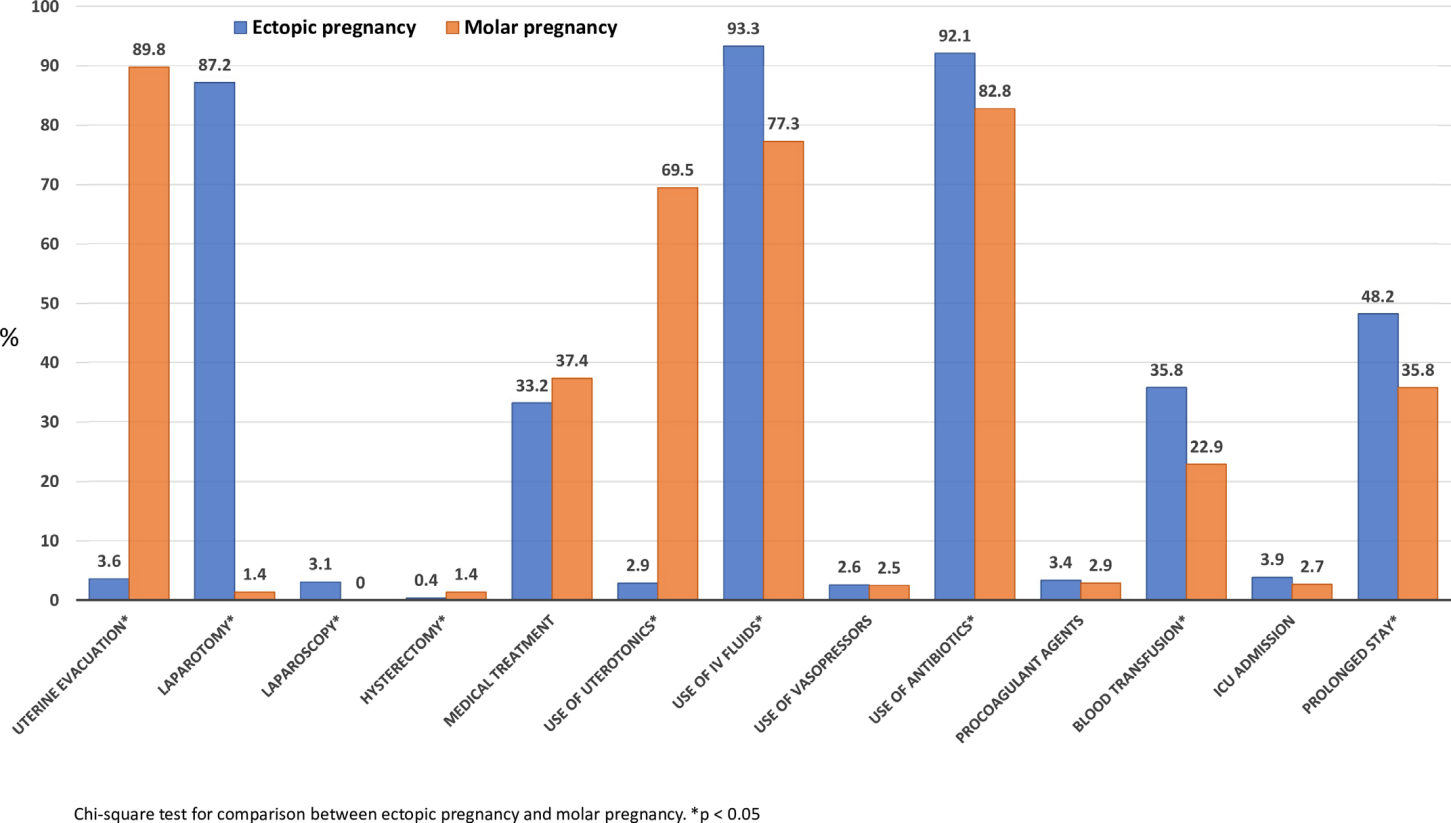
Types of management. Ectopic pregnancy (n=1904) and molar pregnancy (n=511).

In terms of the type of management provided, we observed that compared with women with MP (n=511), those with EP (n=1904) more frequently received interventions such as blood transfusion (35.8% vs 22.9%), laparotomy (87.2% vs 1.4%), laparoscopy (3.1% vs 0.0%), prolonged hospitalisation (48.2% vs 35.8%), use of intravenous fluids (93.3% vs 77.3%) and use of antibiotics (92.1% vs 82.8%). The cases of MP were more frequently managed by hysterectomy (1.4% vs 0.4%) or some type of uterine evacuation (89.8% vs 3.6%). In addition, women with MP received more uterotonics (69.5%) in their treatment than cases of EP. More details are shown in figure 2 and onlinesupplemental tables 4  5.

Among women with EP (n=1904), over 80% received a laparotomy and this was higher in Africa than in LAC (88.9% vs 82.2%, p<0.01). Among women with EP and MP, over 30% received some form of medical treatment for it; however, there were no significant differences in the use of medical treatment for EP between the two regions (32.3% in Africa and 35.6% in LAC). Among women with EP in Africa, our results show higher use of antibiotics (97.2 vs 77.2 %), blood transfusions (41.7 vs 18.9 %) and prolonged facility stay (52.2 vs 36.5%). Although among women with MP (n=511), over 80% received uterine evacuation; however, there was no significant difference between the two regions. Among women with MP, the use of medical treatment was more frequent in LAC (46.4% vs 34.0%); however, the use of uterotonics (74.9% vs 55%), antibiotics (91.1% vs 60.7%) and blood transfusion (27.2% vs 11.4%) were more frequent in Africa (table 2).

**Table 2:** Types of management for EP and MP according to region

Types of management	EP		Total	P value*	MP		Total	P value*
	Africa (n=1411)	LAC (n=493)	(n=1904)		Africa (n=371)	LAC (n=140)	(n=511)	
	n (%)	n (%)	n (%)		n (%)	n (%)	n (%)	
Surgical treatment†								
Uterine evacuation^a^	25 (1.8)	44 (8.9)	69 (3.6)	<0.01	331 (89.2)	128 (91.4)	459 (89.8)	0.46
Laparotomy	1255 (88.9)	405 (82.1)	1660 (87.2)	<0.01	7 (1.9)	0	7 (1.4)	0.2
Laparoscopy	19 (1.3)	41 (8.3)	60 (3.2)	<0.01	0	0	0 (0)	–
Hysterectomy	4 (0.3)	4 (0.8)	8 (0.4)	0.21	6 (1.6)	1 (0.7)	7 (1.4)	0.68
Clinical treatment†								
Medical treatment^a^‡	456 (32.3)	175 (35.6)	631 (33.1)	0.18	126 (34.0)	65 (46.4)	191 (37.4)	0.009
Use of uterotonics^a^	44 (3.1)	11 (2.2)	55 (2.9)	0.31	278 (74.9)	77 (55)	355 (69.5)	<0.01
Use of intravenous fluids^a^	1306 (92.6)	470 (95.5)	1776 (93.3)	0.02	275 (74.1)	120 (85.7)	395 (77.3)	<0.01
Use of vasopressors^a^	40 (2.8)	10 (2)	50 (2.6)	0.33	12 (3.2)	1 (0.7)	13 (2.5)	0.12
Use of antibiotics^a^	1372 (97.2)	380 (77.2)	1752 (92)	<0.01	338 (91.1)	85 (60.7)	423 (82.8)	<0.01
Procoagulant agents^a^	56 (4)	9 (1.8)	65 (3.4)	0.02	14 (3.8)	1 (0.7)	15 (2.9)	0.08
Blood transfusion^a^	589 (41.7)	93 (18.9)	682 (35.8)	<0.01	101 (27.2)	16 (11.4)	117 (22.9)	<0.01
Intensive care unit admission	61 (4.3)	13 (2.6)	74 (3.9)	0.09	13 (3.5)	1 (0.7)	14 (2.7)	0.12
Prolonged facility stay^b^§	737 (52.2)	179 (36.5)	916 (48.1)	<0.01	139 (37.5)	44 (31.4)	183 (35.8)	0.20

Missing data for ectopic pregnancy— – a: 1 b: 2; **prolonged facility stay; .

* Chi-square χ2 test. Comparison between cases of the same disease (EP or MP), considering the region where the case was treated.

† Because women could receive more than one surgical or clinical treatment, totals do not add upBecause women could receive more than one surgical or clinical treatment, totals are not mutually exclusive.

‡ Includes methotrexate or another similar form for molar or ectopic pregnanciesIncludes methotrexate or another similar form for molar or ectopic pregnancies.

§ Prolonged facility stay ≥3 days.

EPectopic pregnancyLACLatin America and the CaribbeanMPmolar pregnancy

Among women with EP, laparotomy was most frequently used among surgical treatments (1660/1904, 87.2%), particularly among women with PLTC (359/379, 94.7%). Among women with EP, the most commonly used clinical treatments were the use of intravenous fluids (1776/1904, 93.3%) and antibiotics (1752/1904, 92%), particularly among women with PLTC for both (fluids=373/398, 98.4%, antibiotics=368/379, 97.1%). Among women with EP, the second most commonly used clinical treatments were a form of medical treatment (631/1904, 33.1%) and blood transfusion (682/1904, 35.4%), where medical treatment was mostly among mild/moderate (495/1410, 35.1%), whereas blood transfusion was mostly among SMOs (94/115, 81.7%). Among women with MP, uterine evacuation was the most frequently used surgical treatment among women with mild/moderate complications (403/449, 89.8%) and PLTC (46/48, 95.8%). Among women with MP, the most commonly used clinical treatments were antibiotics (423/511, 82.8%) followed by intravenous fluids (395/511, 77.3%) and uterotonics (355/511, 69.5%), particularly among PLTC (antibiotics=47/48, 97.9%; fluids=43/48, 89.6%; uterotonics 34/48, 70.8%). Detailed information is shown in online supplemental table 6.

When analysing the type of management according to the PAC score, among women with EP, we observed that facilities with high facility infrastructure (FIS) used a form of medical treatment more frequently (559/1606, 34.9%) than facilities with low (4/15, 26.7%) and intermediate (68/267, 25.5%) FIS. Facilities with intermediate FIS had higher use of uterotonics (17/267, 6.4%) and admission to the ICU (23/267, 8.6%) than facilities with low and high FIS. Considering the SCPAC, we observed that health facilities with higher SCPAC performed laparoscopy more frequently, however, with low prevalence (59/1523, 3.9%), when compared with laparotomy (1308/1523, 85.9%). Furthermore, the use of a form of medical treatment was more frequent in facilities with higher SCPAC. Among women with MP, there was a higher frequency of laparotomy (4/92, 4.3%) and use of uterotonics (76/92, 82.6%) in health facilities with intermediate FIS. Prolonged facility stay was more frequent (159/406, 39.2%) in facilities with high infrastructure scores. In MP cases, the use of uterotonics was more frequent in facilities with intermediate ECPAC (226/289, 78.2%). The use of antibiotics and prolonged facility stay were more frequent in facilities with intermediate and high ECPAC. Detailed information is shown in online supplemental table 7.

## Discussion

This study quantifies the prevalence and management of EP and MP according to severity across 17 LMIC countries in Africa and LAC. Of a total 24 424 women enrolled in the MCS-A study, 7.8% reported an EP and 2.1% an MP. Among both MP and EP, over 70% of conditions were mild/moderate; however, there was still a significant number of women experiencing PLTC among EP (19.9%) and MP (9.4%) and SMO among EP (6%) and MP (3%).

Across various studies on EP and MP, there is a paucity of data on these conditions. Globally, the EP age-standardised disability adjusted life-years (DALYs) have increased by 0.5% in Africa and 4.5% in Latin America.^2^ There have been various individual country studies across Africa, but the estimates vary. In two studies, key significant risk factors for EP were described but they did not specify an overall prevalence.^24 25^ In Cameroon, one study reported an EP prevalence of 1.4%, highlighting a rising trend over recent decades,^26^ whereas another study in Nigeria reported 17.5% of women reporting EP.^27^ Another study conducted in China showed that the prevalence of EP was around 2.5% in 2004 and exhibited an overall decreasing trend from 2011 to 2020.^28^ There is only one study that provides estimates on MP at around 1.9%, which is similar to our findings.^27^ Limited epidemiological data on EP and MP underscores the importance of standardised measurement across different regions. With the use of a single, standardised data collection, we were able to establish a prevalence estimate across 17 countries.

There is limited evidence that quantifies the severity and complications related to EP and MP, as most studies focus on case-fatality rates. Furthermore, more severe outcomes (PLTC, SMO) were prevalent among women with EP compared with those with MP. In EP, 29% of conditions were related to severe outcomes such as PLTC and SMO, compared with 13.6% (9.4% in Africa and 4.3% in Latin America) observed in our main findings related to abortion-related complications.^20 21^ For example, the case-fatality rate for EP was 0.5%, lower than previously reported in less developed countries. A review of the literature that included data collected between 1967 and 1995 reported that in developing African countries, most studies based on facility data reported a fatality rate of 1 to 3% in cases of EP.^29^ More recently, two studies conducted in Nigeria reported a 1.4% case-fatality rate.^30 31^ Other studies from referral facilities in Ghana and Papua New Guinea reported no mortality but acknowledged the morbidity associated with EP.^32 33^ In Papua New Guinea 43% of women had macroscopic evidence of pelvic infection.^33^ In other settings, the frequency of complications in MP was as high as 75%.^34^ Our findings highlighted a disparity in the severity of EP and MP complications compared with high-income countries, where lower case-fatality rates and fewer complications are documented around less than 0.1%.^35 36^

In terms of management, over 90% of EP cases underwent surgical treatment, predominantly laparotomy and 30% received some form of medical treatment (methotrexate or another equivalent form). Facilities with higher PAC capacity used laparoscopy more frequently, though still in a minority of cases. The surgical findings align with those from other regions, such as Cameroon, where 97.6% of EP cases were treated with laparotomy.^26^ A Cochrane review outlined that laparoscopy has advantages over laparotomy, including reduced blood loss, shorter surgery time, faster recovery and shorter hospitalisation, contributing to lower morbidity and mortality^37^; however, access to equipment and training requires resources. Only 3% of EP cases were treated with uterine evacuation, an approach used in specific cases such as cervical or caesarean scar pregnancies.^38^,^40^ Methotrexate is a non-surgical treatment for EP management in patients who are haemodynamically stable, have lower serum hCG levels, small unruptured masses and no foetal cardiac activity on ultrasound, all indicative of early EP diagnosis.^37 41^ Methotrexate can also be used prophylactically in MP cases at high risk of progressing to gestational trophoblastic neoplasia (GTN).^42 43^ Most women with MP underwent uterine evacuation (90%) and received some uterotonic (69.5%). Medications like prostaglandins are not usually used for cervical ripening because they delay the procedure, cause side effects, may increase complication risks and offer no proven benefits.^44^ Excessive vaginal bleeding can occur with surgical management of MP. There is concern about using oxytocic agents like oxytocin and misoprostol routinely, as they might spread trophoblastic tissue through the veins. However, if a life-threatening haemorrhage or ongoing bleeding occurs, uterotonic agents may be used.^42 43^ Although we cannot ascertain the specific clinical conditions of women receiving uterotonics in our study, the disparity in the provision of good quality care highlights the need to strengthen the development and implementation of evidence-based recommendations for EP and MP. This includes comprehensive training for health workers on up-to-date management methods to ensure they can provide the highest standard of care.^45^

In our study, we observed that EP presented at lower gestational ages (less than 12 weeks), likely due to several contributing factors. Usually, EPs begin to have symptoms between 6 and 8 weeks, which can progress to tubal rupture and intra-abdominal haemorrhage.^46^ Increased awareness and screening protocols among healthcare workers and women may have led to earlier medical consultation when symptoms like pelvic pain and bleeding occurred given that we saw that about 90% of women experienced abdominal pain, and 50% showed signs of peritoneal irritation at admission. In other studies, ruptured EP is diagnosed in 15–35% of cases.^12 47^ Studies in Africa and Latin America show high rates of ruptured EP at hospital admission, with 71.3% in Ghana,^32^ 63.3% in Ethiopia^48^ and 40.3% in a Brazilian university hospital.^49^ As molar pregnancies usually do not cause intra-abdominal haemorrhage, the diagnosis may be made later if vaginal bleeding is not investigated, mainly through pelvic ultrasound.^16^

This study is unique in its multi-country, multi-centre approach, encompassing many cases from 17 countries in Africa and LAC. Unlike most previous studies, which focus on individual countries or high-income settings, this research offers a broader perspective on EP and MP in LMICs, providing valuable insights into the prevalence, treatment and outcomes of these conditions across diverse healthcare environments. The inclusion of diverse healthcare settings enhances the generalizability of the findings. However, we acknowledge that our study has its limitations. As a cross-sectional study, we cannot establish cause-effect relationships. Although MP had fewer complications before discharge, lack of follow-up means long-term issues like GTN were not assessed. Furthermore, variations in treatment protocols across countries may have led to inconsistencies in management outcomes, specifically around medical treatment whereby indications of methotrexate or another form were not specified. Data were gathered from medical records, risking missing or erroneous information. Additionally, we used a score that has not yet been validated to assess facility capability, and there may be other methods to evaluate this.

## Conclusion

In Africa and LAC, EP and MP cause significant maternal morbidity and mortality. However, the lack of regional and global data limits our understanding of the burden and severity. Across 17 countries, our study demonstrates that the majority of EP- and MP-associated complications are mild to moderate, further investigation is warranted to address the burden of more severe complications globally. Additionally, the disparity in the provision of good quality care highlights the need to strengthen the implementation of evidence-based recommendations in the clinical and surgical management of EP and MP.

## supplementary material

10.1136/bmjopen-2024-086723 online supplemental file 1

10.1136/bmjopen-2024-086723 online supplemental file 2

10.1136/bmjopen-2024-086723 online supplemental file 3

10.1136/bmjopen-2024-086723 online supplemental file 4

10.1136/bmjopen-2024-086723 online supplemental file 5

10.1136/bmjopen-2024-086723 online supplemental file 6

10.1136/bmjopen-2024-086723 online supplemental file 7

10.1136/bmjopen-2024-086723 online supplemental file 8

## Data Availability

Data are available upon reasonable request.
