# Supplementary material for: Prevalence and management of ectopic and molar pregnancies in 17 countries in Africa and Latin America and the Caribbean: a secondary analysis of the WHO multi-country cross-sectional survey on abortion
Source: BMJ Open. 2024 Oct 14;14(10):e086723. doi: 10.1136/bmjopen-2024-086723 (PMC11474897; doi:10.1136/bmjopen-2024-086723)
Supplement: online supplemental file 2 [file bmjopen-14-10-s002.pdf]

Supplemental table 1. Post abortion care signal functions for facility score

Categories	Signal functions	Maximum score available per facility
Facility general capability: Facility Infrastructure (FIS)	• Electricity available and functioning<br>• Generator available and functioning<br>• Refrigerator available and functioning<br>• Telephone/radio call available and functioning<br>• Email/internet available and functioning<br>• Incinerator available and functioning<br>• Ambulance available and functioning<br>• Water supply available and functioning<br>• Sewerage system available and functioning	9
Capability to provide standard comprehensive postabortion care (SCPAC)	• Removal of retained products available<br>• Parenteral antibiotics available<br>• Uterotonics available (oxytocin or misoprostol)<br>• Intravenous fluids available<br>• Blood transfusion available<br>• 3+ contraceptives offered<br>• 1+ long-acting<br>• modern contraceptive(s) offered<br>• 1+ obstetrician on duty 24/7	8
Extended capability to provide comprehensive postabortion care (ECPAC)	• *Comprehensive indicators* +<br>• At least one guideline currently in use<br>• Clinical audits currently in use<br>• Adult intensive care unit available and functioning<br>• Ultrasound services available and functioning<br>• Biochemical/clinical laboratories available and functioning<br>• 1+ anesthesiologist on duty 24/7	14
