# Supplementary material for: Prevalence and management of ectopic and molar pregnancies in 17 countries in Africa and Latin America and the Caribbean: a secondary analysis of the WHO multi-country cross-sectional survey on abortion
Source: BMJ Open. 2024 Oct 14;14(10):e086723. doi: 10.1136/bmjopen-2024-086723 (PMC11474897; doi:10.1136/bmjopen-2024-086723)
Supplement: online supplemental file 3 [file bmjopen-14-10-s003.pdf]

Supplemental table 2. Distribution of severity of complications for ectopic and molar pregnancies by region, facility infrastructure, health facilities´ capability to provide PAC, clinical and sociodemographic characteristics.

	**Ectopic pregnancies (N=1904)**				**Molar Pregnancies (N=511)**			
	**Mild/moderate complication**	**PLTC**	**SMO**	**Total**	**Mild/moderate complication**	**PLTC**	**SMO**	**Total**
	**n(%)**	**n(%)**	**n(%)**	**n**	**n(%)**	**n(%)**	**n(%)**	**n**
**Total**	1410 (74.1)	379 (**19.9**)	115 (**6.0**)	1904	449 (**87.9**)	48 (9.4)	14 (2.7)	511
**Region§**								
Africa	985 (69.8)	**326 (23.1)**	**100 (7.1)**	1411	**316 (85.2)**	43 (11.6)	12 (3.2)	371
Latin America	425 (86.2)	**53 (10.8)**	15 (3.0)	493	**133 (95.0)**	5 (3.6)	2 (1.4)	140
**Facility infrastructure score**								
Low	11 (73.3)	4 (26.7)	0 (0.00)	15	8 (80)	2 (20)	0 (0.00)	10
Intermediate	182 (68.2)	**57 (21.3)**	28 (10.5)	267	**82 (89.1)**	6 (6.5)	4 (4.4)	92
High	1201 (74.8)	**318 (19.8)**	**87 (5.4)**	1606	**357 (87.9)**	40 (9.9)	9 (2.2)	406
**Standard comprehensive capability for PAC score**								
Low	62 (86.1)	9 (12.5)	1 (1.4)	72	12 (70.6)	3 (17.6)	2 (11.8)	17
Intermediate	670 (77.3)	150 (17.3)	47 (5.4)	867	95 (85.6)	11 (9.9)	5 (4.5)	111
High	678 (70.3)	**220 (22.8)**	**67 (6.9)**	965	**342 (89.3)**	34 (8.9)	7 (1.8)	383
**Extended comprehensive capability for PAC score**								
Low	**32 (91.4)**	3 (8.6)	0 (0.00)	35	7 (63.6)	**3 (27.3)**	1 (9.1)	11
Intermediate	970 (72.5)	**279 (20.9)**	**88 (6.6)**	1337	**282 (87.6)**	31 (9.6)	9 (2.8)	322
High	408 (76.7)	**97 (18.2)**	27 (5.1)	532	**160 (89.9)**	14 (7.9)	4 (2.2)	178
**Age**								
<20	71 (71)	22 (22)	7 (7)	100	59 (84.3)	9 (12.9)	2 (2.9)	70
20-24	308 (72.5)	**88 (20.7)**	**29 (6.8)**	425	**113 (86.9)**	13 (10)	4 (3.1)	130
25-29	423 (74.5)	**115 (20.2)**	**30 (5.3)**	568	**86 (88.7)**	9 (9.3)	2 (2.1)	97

30-34	359 (77)	**82 (17.6)**	**25 (5.4)**	466	**64 (91.4)**	5 (7.2)	1 (1.4)	70
≥35	234 (71.8)	**69 (21.2)**	**23 (7)**	326	**118 (87.4)**	12 (8.9)	5 (3.7)	135
**Cohabitation status**								
With partner	964 (74.4)	**252 (19.4)**	**80 (6.2)**	1296	**343 (86)**	43 (10.8)	13 (3.2)	399
Without partner	345 (73.2)	**101 (21.4)**	**25 (5.3)**	471	**84 (93.3)**	5 (5.6)	1 (1.1)	90
**Number of previous births**								
0	125 (77.1)	27 (16.7)	10 (6.2)	162	18 (85.7)	2 (9.5)	1 (4.8)	21
1-2	718 (76.2)	**171 (18.2)**	**53 (5.6)**	942	**163 (91.1)**	15 (8.4)	1 (0.5)	179
>2	247 (66.9)	**91 (24.7)**	**31 (8.4)**	369	**165 (88.2)**	16 (8.6)	6 (3.2)	187
**Gestational age**								
< 12	953 (76.3)	**235 (18.8)**	**61 (4.9)**	1249	**107 (92.2)**	8 (6.9)	1 (0.9)	116
≥ 12	105 (71.4)	**29 (19.7)**	**13 (8.9)**	147	**196 (84.5)**	27 (11.6)	9 (3.9)	232
**Education**								
No education	95 (58.3)	**45 (27.6)**	**23 (14.1)**	163	**96 (90.6)**	10 (9.4)	0 (0)	106
Primary/Secondary	741 (75.5)	**180 (18.4)**	**60 (6.1)**	981	**230 (87.1)**	26 (9.9)	8 (3)	264
Tertiary	254 (76.5)	**65 (19.6)**	**13 (3.9)**	332	**38 (95)**	2 (5)	0 (0)	40

SMO= Severe Maternal Outcomes; PLTC= Potentially Life-Threatening Complications; PAC= post-abortion care
Chi-square test for comparison between ectopic pregnancy and molar pregnancy. Bold values: $p < 0.05$
[§]Participating countries for Africa: Benin, Burkina Faso, Chad, Democratic Republic of the Congo, Ghana, Kenya, Malawi, Mozambique, Niger, Nigeria, Uganda; participating countries for LAC: Argentina, Bolivia, Brazil, Dominican Republic, El Salvador and Peru
