# Supplementary material for: Prevalence and management of ectopic and molar pregnancies in 17 countries in Africa and Latin America and the Caribbean: a secondary analysis of the WHO multi-country cross-sectional survey on abortion
Source: BMJ Open. 2024 Oct 14;14(10):e086723. doi: 10.1136/bmjopen-2024-086723 (PMC11474897; doi:10.1136/bmjopen-2024-086723)
Supplement: online supplemental file 4 [file bmjopen-14-10-s004.pdf]

Supplemental table 3. Symptoms and signs at hospital admission

	**Ectopic pregnancy n (%)**		**Molar pregnancy (%)**	
	**Africa**	**Latin America**	**Africa**	**Latin America**
**Hospital admission – first assessment**				
Vaginal bleeding[a,c]	879 (65.5)	323 (67.7)	284 (83.3)	107 (79.9)
Abdominal pain[a]	1252 (93.3)	416 (87.2)		
Abnormal hCG levels[a]	263 (19.6)	187 (39.2)		
Referred for suspicious of ectopic pregnancy[a]	709 (52.8)	211 (44.2)		
Collapsed/fainted[a]	182 (13.6)	43 (9)		
Uterine size greater than dates[c]			178 (52.2)	78 (58.2)
Vomiting[c]			93 (27.3)	25 (18.7)
Referred for suspicious of molar pregnancy[c]			94 (27.6)	91 (67.9)
Abdominal rebounding[b]	748 (53)	199 (40.4)	6 (1.6)	7 (5)
Abdominal distension[b]	344 (24.4)	49 (10)	40 (10.8)	4 (2.9)
Abdominal tension[b]	426 (30.2)	47 (9.6)	19 (5.1)	0 (0)
Abdominal tenderness[b]	1173 (83.1)	367 (74.6)	85 (22.9)	34 (24.3)
**Hospital admission (assessment and outcomes throughout hospital stay)**				
Vaginal bleeding < 24 hs[b,d]	610 (43.2)	175 (35.6)	221 (59.7)	48 (34.3)
Vaginal bleeding > 24 hs[b,d]	7 (0.5)	3 (0.6)	13 (3.5)	3 (2.1)
Intra-abdominal injury < 24 hs[b]	615 (43.6)	225 (45.7)	44 (11.9)	14 (10)
Intra-abdominal injury > 24 hs[b]	8 (0.6)	8 (1.6)	0 (0)	0 (0)
Infection < 24 hs[b]	72 (5.1)	6 (1.2)	26 (7)	4 (2.9)
Infection > 24 hs[b]	7 (0.5)	1 (0.2)	4 (1.1)	0 (0)

Missing data ectopic pregnancy: a: 85 b:1
Missing data molar pregnancy: c: 36 d: 1
