# Supplementary material for: Prevalence and management of ectopic and molar pregnancies in 17 countries in Africa and Latin America and the Caribbean: a secondary analysis of the WHO multi-country cross-sectional survey on abortion
Source: BMJ Open. 2024 Oct 14;14(10):e086723. doi: 10.1136/bmjopen-2024-086723 (PMC11474897; doi:10.1136/bmjopen-2024-086723)
Supplement: online supplemental file 5 [file bmjopen-14-10-s005.pdf]

Supplemental table 4. Types of management among women with ectopic and molar pregnancies.

**Types of management**	**Ectopic pregnancy** (%)	**Molar pregnancy** (%)	**Total**	**p-value**
Surgical treatment§				
Uterine evacuation	69 (3.6)	459 (89.8)		<0.001
Laparotomy	1660 (87.2)	7 (1.4)	1667	<0.001
Laparoscopy	60 (3.1)	0	60	<0.001
Hysterectomy	8 (0.4)	7 (1.4)	15	0.02
Clinical treatment§				
Medical treatment[β]	631 (33.2)	191 (37.4)	822	0.07
Use of uterotonics	55 (2.9)	355 (69.5)	410	<0.001
Use of IV fluids	1776 (93.3)	395 (77.3)	2171	<0.001
Use of vasopressors	50 (2.6)	13 (2.5)	63	0.91
Use of antibiotics	1752 (92.1)	423 (82.8)	2175	<0.001
Procoagulant agents	65 (3.4)	15 (2.9)	80	0.59
Blood transfusion	682 (35.8)	117 (22.9)	799	<0.001
ICU admission	74 (3.9)	14 (2.7)	88	0.21
Prolonged stay	916 (48.2)	183 (35.8)	1099	<0.001

Chi-square test for comparison between ectopic pregnancy and molar pregnancy.

[β]Includes methotrexate or another similar form for molar or ectopic pregnancies

[§]Because women could receive more than one surgical or clinical treatment, totals do not add up
