# Supplementary material for: Prevalence and management of ectopic and molar pregnancies in 17 countries in Africa and Latin America and the Caribbean: a secondary analysis of the WHO multi-country cross-sectional survey on abortion
Source: BMJ Open. 2024 Oct 14;14(10):e086723. doi: 10.1136/bmjopen-2024-086723 (PMC11474897; doi:10.1136/bmjopen-2024-086723)
Supplement: online supplemental file 6 [file bmjopen-14-10-s006.pdf]

Supplemental table 5. Types of uterotonics among women with ectopic and molar pregnancies.

**Types of uterotonics**	**Ectopic pregnancy** (%)	**Molar pregnancy** (%)	**Total**
Oxytocin only	36 (65.4)	128 (36.1)	164
Misoprostol and oxytocin	3 (5.45)	92 (25.9)	95
Misoprostol only	8 (14.5)	79 (22.2)	87
Misoprostol / Oxytocin / Methylergonovine / Ergometrine	1 (1.8)	22 (6.2)	23
Oxytocin / Methylergonovine / Ergometrine	2 (3.6)	19 (5.3)	21
Misoprostol / Methylergonovine / Ergometrine	2 (3.6)	8 (2.2)	10
Methylergonovine / Ergometrine	1 (1.8)	7 (2.0)	8
Other / Unknown	2 (3.6)	0 (0)	2
Total	55 (100)	355 (100)	410
