# Supplementary material for: Prevalence and management of ectopic and molar pregnancies in 17 countries in Africa and Latin America and the Caribbean: a secondary analysis of the WHO multi-country cross-sectional survey on abortion
Source: BMJ Open. 2024 Oct 14;14(10):e086723. doi: 10.1136/bmjopen-2024-086723 (PMC11474897; doi:10.1136/bmjopen-2024-086723)
Supplement: online supplemental file 7 [file bmjopen-14-10-s007.pdf]

Supplemental table 6. Types of management for EP and MP by severity of complication.

**Types of management**	**Ectopic pregnancy**			**Total (N=1904)**	**p-value***	**Molar pregnancy**			**Total (N=511)**	**p-value***
	Mild/moderate n(%) (N=1410)	PLTC n(%) (N=379)	SMO n(%) (N=115)			Mild/moderate n(%) (N=449)	PLTC n(%) (N=48)	SMO n(%) (N=14)		
Surgical treatment[§]										
Uterine evacuation[a]	52 (3.7)	11 (2.9)	6 (5.2)	69 (3.6)	0.49	403 (89.8)	46 (95.8)	10 (71.4)	459 (89.8)	0.03
Laparotomy	1197 (84.9)	359 (94.7)	104 (90.4)	1660 (87.2)	<0.01	4 (0.9)	1 (2.1)	2 (14.3)	7 (1.4)	<0.01
Laparoscopy	51 (3.6)	8 (2.1)	1 (0.9)	60 (3.2)	0.11	-	-	-	0 (0)	-
Hysterectomy	4 (0.3)	2 (0.5)	2 (1.7)	8 (0.4)	0.07	5 (1.1)	0 (0)	2 (14.3)	7 (1.4)	0.01
Clinical treatment[§]										
Medical treatment [a,β]	495 (35.1)	105 (27.7)	31 (27)	631 (33.1)	<0.01	169 (37.6)	18 (37.5)	4 (28.6)	191(37.4)	0.78
Use of uterotonics[a]	36 (2.6)	11 (2.9)	8 (7)	55 (2.9)	0.02	314 (69.9)	34 (70.8)	7 (50)	355 (69.5)	0.27
Use of IV fluids[a]	1298 (92.1)	373 (98.4)	105 (91.3)	1776 (93.3)	<0.01	340 (75.7)	43 (89.6)	12 (85.7)	395 (77.3)	0.07
Use of vasopressors[a]	21 (1.5)	12 (3.2)	17 (14.8)	50 (2.6)	<0.01	9 (2)	1 (2.1)	3 (21.4)	13 (2.5)	<0.01
Use of antibiotics[a]	1275 (90.5)	368 (97.1)	109 (94.8)	1752 (92)	<0.01	363 (80.8)	47 (97.9)	13 (92.9)	423 (82.8)	<0.01
Procoagulant agents[a]	32 (2.3)	19 (5)	14 (12.2)	65 (3.4)	<0.01	12 (2.7)	3 (6.2)	0 (0)	15 (2.9)	0.35

Blood transfusion[a]	290 (20.6)	298 (78.6)	94 (81.7)	682 (35.8)	<0.01	77 (17.1)	34 (70.8)	6 (42.9)	117 (22.9)	<0.01
ICU admission	31 (2.2)	28 (7.4)	15 (13)	74 (3.9)	<0.01	9 (2)	2 (4.2)	3 (21.4)	14 (2.7)	<0.01
Prolonged facility stay[b,**]	625 (44.4)	214 (56.5)	77 (67)	916 (48.1)	<0.01	149 (33.2)	27 (56.2)	7 (50)	183 (35.8)	<0.01

SMO= Severe Maternal Outcomes; PLTC= Potentially Life-Threatening Complications

*Chi-square test. Comparison between cases of the same disease (EP or MP), considering the severity of the complications.

Missing data for ectopic pregnancy – a:1 b: 2; **prolonged hospital stay ≥3 days

[β]Includes methotrexate or another similar form for molar or ectopic pregnancies

[§]Because women could receive more than one surgical or clinical treatment, totals do not add up
