# Supplementary material for: Prevalence and management of ectopic and molar pregnancies in 17 countries in Africa and Latin America and the Caribbean: a secondary analysis of the WHO multi-country cross-sectional survey on abortion
Source: BMJ Open. 2024 Oct 14;14(10):e086723. doi: 10.1136/bmjopen-2024-086723 (PMC11474897; doi:10.1136/bmjopen-2024-086723)
Supplement: online supplemental file 8 [file bmjopen-14-10-s008.pdf]

Supplemental table 7. Types of management in EP and MP by health facility's infrastructure and capability to provide post abortion care.

	Ectopic pregnancy (N=1904)									Molar pregnancy (N=511)								
	FIS N=1888			SCPAC N=1904			ECPAC N=1904			FIS N=508			SCPAC N=511			ECPAC N=511		
**Types of management**	**Low (%) N=15**	**Intermediate (%) N=267**	**High (%) N=1606**	**Low (%) N=35**	**Intermediate (%) N=346**	**High (%) N=1523**	**Low (%) N=35**	**Intermediate (%) N=1007**	**High (%) N=862**	**Low (%) N=10**	**Intermediate (%) N=92**	**High (%) N=406**	**Low (%) N=13**	**Intermediate (%) N=66**	**High (%) N=432**	**Low (%) N=13**	**Intermediate (%) N=289**	**High (%) N=209**
Surgical treatment §																		
Uterine evacuation[a,d,f]	0 (0)	8 (3)	61 (3.8)	0 (0)	15 (4.3)	54 (3.5)	0 (0)	29 (2.9)	40 (4.6)	7 (70)	81 (88)	368 (90.6)	11 (84.6)	63 (95.4)	385 (89.1)	11 (84.6)	265 (91.7)	183 (87.6)
Laparotomy[b,f]	11 (73.3)	235 (88)	1398 (87)	32 (**91.4**)	320 (**92.5**)	1308 (85.9)	32 (**91.4**)	894 (**88.8**)	734 (85.1)	0 (0)	4 (**4.3**)	3 (0.7)	0 (0)	1 (1.5)	6 (1.4)	0 (0)	6 (2.1)	1 (0.5)
Laparoscopy[b,f]	0 (0)	5 (1.9)	55 (3.4)	0 (0)	1 (0.3)	59 (**3.9**)	0 (0)	16 (1.6)	44 (**5.1**)	-	-	-	-	-	-	-	-	-
Hysterectomy[b,f]	0 (0)	0 (0)	8 (0.5)	0 (0)	1 (0.3)	7 (0.5)	0 (0)	3 (0.3)	5 (0.6)	0 (0)	3 (3.3)	4 (1)	0 (0)	3 (4.5)	4 (0.9)	0 (0)	6 (2.1)	1 (0.5)
Clinical treatment §																		
Medical treatment β[a,d,f]	4 (26.7)	68 (25.5)	559 (**34.9**)	6 (17.1)	85 (**24.6**)	540 (**35.5**)	6 (17.1)	382 (**38**)	243 (**28.2**)	3 (30)	38 (41.3)	148 (36.4)	6 (46.1)	17 (25.8)	168 (38.9)	6 (46.1)	113 (39.1)	72 (34.4)
Use of uterotonics[a,d,f]	0 (0)	17 (**6.4**)	38 (2.4)	0 (0)	7 (2)	48 (3.1)	0 (0)	38 (**3.8**)	17 (2)	6 (60)	76 (**82.6**)	272 (67)	8 (61.5)	51 (77.3)	296 (68.5)	8 (61.5)	226 (**78.2**)	121 (57.9)
Use of IV fluids[a,d,f]	13 (86.7)	257 (96.2)	1490 (92.8)	34 (97.1)	332 (95.9)	1410 (92.6)	34 (97.1)	944 (93.7)	798 (92.7)	6 (60)	73 (79.3)	313 (77.1)	11 (84.6)	45 (68.2)	339 (78.5)	11 (**84.6**)	206 (71.3)	178 (**85.2**)
Use of vasopressors[a,d,f]	0 (0)	8 (3.0)	42 (2.6)	0 (0)	8 (2.3)	42 (2.8)	0 (0)	29 (2.9)	21 (2.4)	0 (0)	2 (2.2)	11 (2.7)	1 (7.7)	3 (4.5)	9 (2.1)	1 (7.7)	10 (3.5)	2 (1)
Use of antibiotics[a,d,f]	14 (93.3)	254 (95.1)	1470 (91.6)	30 (85.7)	322 (93.1)	1400 (92)	30 (85.71)	970 (**96.3**)	752 (87.3)	6 (60)	79 (85.9)	337 (83)	8 (61.5)	52 (78.8)	363 (84)	8 (61.5)	258 (**89.3**)	157 (**75.1**)
Procoagulant agents[a,d,f]	0 (0)	4 (1.5)	61 (3.8)	0 (0)	15 (4.3)	50 (3.3)	0 (0)	40 (4)	25 (2.9)	0 (0)	0 (0)	15 (3.7)	0 (0)	3 (4.5)	12 (2.8)	0 (0)	11 (3.8)	4 (1.9)
Blood transfusion[a,d,f]	2 (13.3)	106 (39.7)	573 (35.7)	3 (8.6)	113 (**32.7**)	566 (**37.2**)	3 (8.6)	371 (**36.8**)	308 (**35.8**)	3 (30)	18 (19.6)	96 (23.6)	4 (30.8)	13 (19.7)	100 (23.1)	4 (30.8)	66 (22.8)	47 (22.5)

ICU admission[b,f]	0 (0)	23 (**8.6**)	51 (3.2)	0 (0)	13 (3.8)	61 (4)	0 (0)	38 (3.8)	36 (4.2)	0 (0)	6 (6.5)	8 (2)	0 (0)	3 (4.5)	11 (2.5)	0 (0)	12 (4.1)	2 (1)
Prolonged facility stay[c,e,f]	6 (40)	143 (53.6)	759 (47.3)	16 (**45.7**)	139 (40.2)	761 (**50**)	16 (45.7)	458 (45.5)	442 (**51.4**)	2 (20)	22 (23.9)	159 (**39.2**)	2 (15.4)	25 (37.9)	156 (36.1)	2 (15.4)	94 (**32.5**)	87 (**41.6**)

FIS= Facility Infrastructure; SCPAC= Standard Comprehensive Capability for PAC; ECPAC= Extended Comprehensive Capability for PAC

Chi-square test with p-value <0.05. Comparison between cases of the same disease (EP or MP), considering the health facility's infrastructure, standard comprehensive capability for PAC score and extended comprehensive capability for PAC score. Bold values: $p < 0.05$

Missing cases FIS ectopic pregnancy: a:17 b:16 c:18

Missing cases SCPAC and ECPAC ectopic pregnancy: d:1 e:2

Missing cases FIS molar pregnancy: f:3

[β]Includes methotrexate or another similar form for molar or ectopic pregnancies

[§]Because women could receive more than one surgical or clinical treatment, totals do not add up
