# Supplementary material for: Prevalence and management of ectopic and molar pregnancies in 17 countries in Africa and Latin America and the Caribbean: a secondary analysis of the WHO multi-country cross-sectional survey on abortion
Source: BMJ Open. 2024 Oct 14;14(10):e086723. doi: 10.1136/bmjopen-2024-086723 (PMC11474897; doi:10.1136/bmjopen-2024-086723)
Supplement: Supplementary file 9 [file bmjopen-2024-086723.reviewer_comments.pdf]

PEER REVIEW HISTORY

BMJ Open publishes all reviews undertaken for accepted manuscripts. Reviewers are asked to complete a checklist review form and are provided with free text boxes to elaborate on their assessment. These free text comments are reproduced below.

ARTICLE DETAILS

Title (Provisional)

Prevalence and management of ectopic and molar pregnancies in 17 countries in Africa and Latin America and the Caribbean: a secondary analysis of the WHO multi-country cross-sectional survey on abortion

Authors

Cavalari, Camila Ayume Amano; Mehrtash, Hedieh; Brizuela, Vanessa; Baguiya, Adama; Adu-Bonsaffoh, Kwame; Cecatti, Jose Guilherme; Bahamondes, Luis; Charles, Charles M'poca; Govule, Philip; Dossou, Jean-Paul; Souza, Renato T; Leão, Luis Henrique; Filippi, Veronique; Tunçalp, Özge; Baccaro, Luiz Francisco

VERSION 1 - REVIEW

**Reviewer**	**1**
**Name**	**Aimagambetova, Gulzhanat**
**Affiliation**	**Nazarbayev University, Clinical Academic Department of Women's Health**
**Date**	**02-May-2024**
**COI**	**no**

Maternal mortality and pregnancy complications are hot topics in OBGYN, especially in LMICs. Thus the topic of the discussion is important. However, the manuscript requires corrections. Comments:

1.Line 6 in the introduction - remove word "hemorrhagic" from the definition. It sounds confusing.

2. The methods part should include the following sections:

Study design (not clearly written), study subjects, and specific methods for this study, ethics statement if applicable.

3.Results are interesting and supported by clear tables and figures.

4. In the discussion part is too long and difficult to comprehend in the current version. It requires restructuring. The following outline is suggested:

Outline for the Discussion part

1.1 Rationale of the study (why it was done)

1.1.1 Main findings of the study

1.1.2 What makes your study unique

1.1.3 What it adds to what we already know

1.2 Study subjects

1.3 Subject of the discussion

Comparison of your results with previous studies in the field. Agreement and disagreement with

the studies compared

1.4 Study strengths and limitations

1.5 Clinical implication

12. The authors performed a very interesting study, and thus the conclusion should be formulated

---

**Reviewer**	**2**
**Name**	**Bonati, Maurizio**
**Affiliation**	**IRCCS- istituto di ricerche farmacologiche "Mario Negri", Public Health**
**Date**	**16-May-2024**
**COI**	**no conflict of interest**

Ectopic pregnancies and molar pregnancy are two conditions widely studied in the northern hemisphere. Protocols and guidelines are available for their monitoring and clinical management. Research is still ongoing. However, all this with little attention and participation for countries with limited resources. Therefore the general purpose of the present work is important. However, in its present form the manuscript is too generic, descriptive but not specific, with little or no proposition for those conditions and situations with limited resources.

The message of “what is known” and “what does this work add” to knowledge and improvement of practice is one that should benefit the reader and the authors to provide.The authors must make it explicit.

"What is known". The bibliography needs to be updated. What is known in the countries involved, although scarce, must be compared with the results obtained. Discussing UK, USA, The Netherland vs Nigeria, Papua New Guinea in terms of prevalence is not very useful,

when the numbers are small, poorly controlled and in completely different healthcare contexts. Where and when possible comparisons over time for the same country involved, between comparable areas and services can also be more informative for planning.

Standardized and internationally recognized guidelines and protocols should represent (for some variables) the "control" with which to analyze the results.

No variables associated with obstetric history were analysed, but it would be useful to document the poor pre-event care and not report it as a possible hypothesis under discussion.

The quality of hospital care and the appropriateness of treatments for EP and MP vary widely even in the North of the world and with the variability reported in the results the improvement in care for MP and MP is universal, although of different magnitude.

"Future research..." the conclusions in the present form are so general that they are suitable always and everywhere. The authors should better express the cognitive and indicative contribution to improving treatment for EP and MP of their work.

VERSION 1 - AUTHOR RESPONSE

Reviewer: 1
Dr. Gulzhanat Aimagambetova, Nazarbayev University
Comments to the Author:

Maternal mortality and pregnancy complications are hot topics in OBGYN, especially in LMICs. Thus the topic of the discussion is important. However, the manuscript requires corrections. Comments:
1.Line 6 in the introduction - remove word "hemorrhagic" from the definition. It sounds confusing.

Response: we removed the word as suggested.

2. The methods part should include the following sections:
Study design (not clearly written), study subjects, and specific methods for this study, ethics statement if applicable.

Response: We have made changes to the methods section as suggested, and now have the following sub-headers: study design and subjects; study measures; and data analysis.

3.Results are interesting and supported by clear tables and figures.

Response: thank you very much

4. In the discussion part is too long and difficult to comprehend in the current version. It requires restructuring. The following outline is suggested:

Outline for the Discussion part
1.1 Rationale of the study (why it was done)
1.1.1 Main findings of the study
1.1.2 What makes your study unique
1.1.3 What it adds to what we already know
1.2 Study subjects
1.3 Subject of the discussion
Comparison of your results with previous studies in the field. Agreement and disagreement with
the studies compared
1.4 Study strengths and limitations
1.5 Clinical implication
12. The authors performed a very interesting study, and thus the conclusion should be formulated

Response: As suggested, we have completely reformulated and shortened the discussion section to ensure that the main findings and takeaway messages are made clearer, that the uniqueness of our study is highlighted, and what our contributions to the evidence on ectopic and molar pregnancies are, including the implications for research and practice. We have also reformulated our strengths and limitations section to respond to this and other reviewer comments.

Reviewer: 2
Dr. Maurizio Bonati, IRCCS- istituto di ricerche farmacologiche "Mario Negri"
Comments to the Author:

Ectopic pregnancies and molar pregnancy are two conditions widely studied in the northern hemisphere. Protocols and guidelines are available for their monitoring and clinical management. Research is still ongoing. However, all this with little attention and participation for countries with limited resources. Therefore the general purpose of the present work is important. However, in its present form the manuscript is too generic, descriptive but not specific, with little or no proposition for those conditions and situations with limited resources.
The message of "what is known" and "what does this work add" to knowledge and improvement of practice is one that should benefit the reader and the authors to provide. The authors must make it explicit.

Response: We thank the reviewer for this comment and have now added more relevant discussion points to highlight what we believe are very relevant and important contributions to the field. We have completely reformulated the discussion section for clarity, succinctness, and relevance in response to this and other reviewer comments. You can find the revised discussion section on pages 13-16 (in tracked changes version).

"What is known". The bibliography needs to be updated. What is known in the countries involved, although scarce, must be compared with the results obtained. Discussing UK, USA, The Netherland vs Nigeria, Papua New Guinea in terms of prevalence is not very useful, when the numbers are small, poorly controlled and in completely different healthcare contexts. Where and when possible comparisons over time for the same country involved, between comparable areas and services can also be more informative for planning.

Response: Thanks for the comment. In fact, the lack of data on EP and MP published in the countries included in the present study was one of the drivers to conducting this analysis. We have reformulated the discussion section to ensure better comparisons are made, especially regarding LMIC and high-income countries. Changes can be found in the second and third paragraphs in the discussion section (pages 13 and 14 in tacked changes version), as follows:

"Across various studies on EP and MP, there is a paucity of data on these conditions. Globally, the EP age-standardized disability adjusted life-years (DALYs) have increased by

0.5% in Africa and 4.5% in Latin America [2]. There have been various individual country studies across Africa, but the estimates vary. In two studies, key significant risk factors for EP were described but they did not specify an overall prevalence [24,25] . In Cameroon, one study reported an EP prevalence of 1.4%, highlighting a rising trend over recent decades [26] while another study in Nigeria reported 17.5% of women reporting EP [27]. Another study conducted in China showed that the prevalence of EP was around 2.5% in 2004 and exhibited an overall decreasing trend from 2011 to 2020 [28]. There is only one study that provides estimates on MP at around 1.9%, which is similar to our findings [27]. Limited epidemiological data on EP and MP underscores the importance of standardized measurement across different regions. With the use of a single, standardised data collection, we were able to establish a prevalence estimate across 17 countries.

There is limited evidence that quantifies the severity and complications related to EP and MP, as most studies focus on case-fatality rates. Furthermore, more severe outcomes (PLTC, SMO) were prevalent among women with EP compared to those with MP. In EP, 29% of conditions were related to severe outcomes such as PLTC and SMO, compared to 13.6% (9.4% in Africa and 4.3% in Latin America) observed in our main findings related to abortion-related complications [20,21]. For example, the case-fatality rate for EP was 0.5%, lower than previously reported in less developed countries. A review of the literature that included data collected between 1967 and 1995 reported that in developing African countries, most studies based on facility data reported a fatality rate of 1 to 3% in cases of ectopic pregnancy [29]. More recently, two studies conducted in Nigeria reported a 1.4% case-fatality rate [30,31]. Other studies from referral facilities in Ghana and Papua New Guinea reported no mortality but acknowledged the morbidity associated with EP [32,33]. In Papua New Guinea 43% percent of women had macroscopic evidence of pelvic infection [33]. In other settings, the frequency of complications in MP was as high as 75% [34]. Our findings highlighted a disparity in the severity of EP and MP complications compared to high-income countries, where lower case-fatality rates and fewer complications are documented around less than 0.1% [35,36].”

Standardized and internationally recognized guidelines and protocols should represent (for some variables) the "control" with which to analyze the results. No variables associated with obstetric history were analysed, but it would be useful to document the poor pre-event care and not report it as a possible hypothesis under discussion.

Response: we have completely reformulated the discussion section, and believe the edits made in response to this and other reviewers now make the arguments much clearer to the reader.

The quality of hospital care and the appropriateness of treatments for EP and MP vary widely even in the North of the world and with the variability reported in the results the improvement in care for EP and MP is universal, although of different magnitude. "Future research..." the conclusions in the present form are so general that they are suitable always and everywhere. The authors should better express the cognitive and indicative contribution to improving treatment for EP and MP of their work.

Response: Thanks for this observation, which prompted us to reformulate the conclusion, as follows:

"In Africa and LAC, EP and MP cause significant maternal morbidity and mortality. However, the lack of regional and global data limits our understanding of the burden and severity. Across 17 countries, our study demonstrates that the majority of EP and MP associated complications are mild to moderate, further investigation is warranted to address the burden of more severe complications globally. Additionally, the disparity in the provision of good quality care highlights the need to strengthen the implementation of evidence-based recommendations in the clinical and surgical management of EP and MP."
